# Human Milk, Environmental Toxins and Pollution of Our Infants: Disturbing Findings during the First Six Months of Life

**Published:** 2006-06

**Authors:** Gerd-Michael Lackmann

**Affiliations:** *Outpatient Paediatric Office, Hamburg, and Department of Paediatrics, Heinrich Heine University, Dusseldorf, Germany*

**Keywords:** breast-feeding, organochlorine compounds, environmental pollutants, dietary toxicology, children

## Abstract

**Background:**

Toxic organochlorine compounds (OC) are transmitted from mother to infant during lactation. OC are ingested by and stored in their offspring. Different harmful effects later in life have been attributed to the body pollution with these OC, although these findings are still discussed in an argumentative manner, since first other investigators could demonstrate beneficial effects of breast-feeding despite elevated OC concentrations, and second the benefits of breast-feeding are an unchallenged fact, especially in those countries, where infant formulas are not available. It was the aim of the present study to determine the lactational uptake of different OC (polychlorinated biphenyls (PCB), hexachlorobenzene (HCB), and DDE) in breast-fed vs. bottle-fed infants up to six months of age.

**Methods:**

With the written informed consent of the parents, blood samples were taken from each ten breast-fed and bottle-fed infants, respectively. The specimens were immediately centrifuged, and serum was stored in glass tubes without an anticoagulant up to analysis. Three higher-chlorinated PCB congeners (IUPAC Nos. 138, 153, and 180), HCB, and DDE, the main metabolite of DDT in mammals, were determined with capillary gas chromatography with electron capture detection. In addition, reliability was tested with gas chromatography-mass spectrometry. Possible correlations of OC with personal data were tested with a standard multivariate regression model. Differences between study groups were tested on mean differences with Wilcoxons test for independent samples.

**Results:**

We could demonstrate that breast-fed infants have significantly (*p*<0.0001) elevated serum concentrations of all OC as early as at the age of six weeks (90%), which over and above nearly doubled further until the age of six months. (Median (μg/L); A=six weeks; B=six months): PCB 138, A: 0.40 vs. 0.09; B: 0.72 vs. 0.07; PCB 153, A: 0.57 vs. 0.11; B: 0.99 vs. 0.09; PCB 180, A: 0.33 vs. 0.04; B: 0.58 vs. 0.02; PCB (sum of the three PCB congeners), A: 1.19 vs. 0.29; B: 2.28 vs. 0.18; HCB, A: 0.13 vs. 0.04; B: 0.43 vs. 0.07; DDE, A: 1.05 vs. 0.18; B: 1.90 vs. 0.19.

**Conclusions:**

The discussion about the benefits of breast-feeding should be reconsidered again, with special emphasis on the question, whether the recommendations for breast-feeding can unreservedly be maintained for the future throughout the world, especially in face of the availability of infant formulas in industrialized vs. Third World countries, respectively.

## INTRODUCTION

Despite the significant decline (up to 90%) of most carcinogenic and teratogenic environmental toxins, like polychlorinated biphenyls (PCBs), hexachlorobenzene (HCB), and 1,1,1-trichloro-2,2-bis (p-chlorophenyl) ethane (DDT), in habitants of developed countries during the past two decades (1), primarily as the result of their prohibition in industrial manufacturing, all those organochlorine compounds (OC) are still present in our biosphere. Their persistence and long half-life is because of their chemical stability and absence of metabolizing enzymes that can catalyze reactions leading to breakdown or conjugation in mammalian organisms. They still add to our food-chain, are transmitted from mother to foetus, and mother’s milk due to its high lipid content as compared with serum and extra-cellular fluid at all is an exceptional accumulation pathway. A woman eliminates up to 10% of her body pollution with PCBs during lactation (2), and these OC are then ingested by and stored in her offspring. So, an exclusively breast-fed infant accumulates 3 to 5% of his life-long body burden with these OC during the first 6 months of his life, which could be demonstrated in serum as well as in fat tissue (3, 4). Therefore, breast-feeding has been held responsible for elevated concentrations of these OC as well as for different harmful effects in children later in life by many studies, as growth retardation in girls (but not in boys, dependent on DDE, but not PCB concentrations) (5), atopic manifestations, including asthma (6), and neurological impairment (4, 7, 8). Indeed, great deal of attention has been paid to the vulnerability of the embryo and the foetus and the breast-fed infant to OC and other possibly harmful agents. Because the embryo and the child are growing and their tissues and organs are differentiating, deleterious effects may occur at lower exposures to some chemicals, drugs, and physical agents and produce more severe effects than those seen in adults (9). On the other hand, it is important to note that children and adolescents have better recuperative capacities than adults for many toxic agents, and, similarly, appropriate drug dosages may be lower or higher on a mg / kg or surface area basis in children than in adults to attain effective therapeutic blood levels or to avoid toxicity (10). In addition, effects produced by OC are not always deleterious or irreversible. This means that for some exposures, the young can recover from some effects more rapidly and completely than adults (9). This explains, why, in contrast to the studies cited above, other investigators could demonstrate, for example, beneficial effects of breast-feeding on neurodevelopment (11), or even heavier and more frequent cases of gastroenteritis or otitis media in bottle-fed than in breast-fed infants (12). This reveals the fundamental complexity of the appraisal whether OC and other environmental toxicants may have detrimental effects to our children, and whether breast-feeding, as a proven source of a high body pollution with OC should be generally recommended for all children throughout the world, thereby having additionally in mind the availability of infant formulas in different countries throughout the world.

Nevertheless, the mechanisms of toxicity and harmful effects later in life following exposure to environmental pollutants prenatally or during infancy are widely not completely understood. Growth retardation may be a general indicator of toxicity and suggests that specific organ systems could be affected. Although DDE is a hormonally active agent, a plausible biological mechanism for an effect on growth is not readily apparent (13). In case of wheezing and asthma, two pathways–immunological and/or hormonal–could be involved in the relationship between pollution with OC and these disorders. The immunologic effects of OC exposure have been suggested by many studies, although its mechanism remains unclear until now (14). Possible explanations are changes in immune cells, immunoglobulins, and cytokines. OC interfere with hormonal receptors and mimic estrogen activity, which might modulate immunologic responses (15). Nevertheless, sexual hormones have been related to asthma by routes other than immunomodulation by unknown mechanisms (16). The effect of OC on brain growth and possible later neurodevelopmental impairment is still an important issue of concern (17). Because multiple variables play important roles in the development of the human brain, it is difficult, if not impossible, to elucidate the interactions and relations between all variables. Neurotoxic exposures that affect subtle brain functioning manifest themselves only, when this functioning is needed, and, therefore, might be never detected (18). The effects of OC on health are most often subtle, because they usually occur at concentrations that are not expected to result in acute toxic symptoms, but these probable small effects at an individual level might have a large impact at the population level (19).

Recently, significantly elevated HCB and DDE concentrations in breast-fed infants could be demonstrated at 1 year of age (20), but, unfortunately, the lactational uptake of harmful OC has not been investigated during the first six months of life until now, the time span, most mothers breast-fed their infants and finish breast-feeding. It was the aim of the present study to determine the lactational uptake of PCBs, HCB, and DDE (1,1-dichloro-2,2-bis(p-chlorophenyl)-ethylene), the main metabolite of DDT in mammals, in breast-fed vs. bottle-fed human infants at the age of 6 weeks and 6 months, respectively. As early as at the age of six weeks of life, breast-fed infants had accumulated about 90% more OC than their bottle-fed peers, as could be demonstrated in a preliminary evaluation of our study’s results obtained at that time (21). Here, we now present the results of our study at the age of six months of life, and the further course of uptake of these harmful OC in the same study population of infants in comparison with the results previously published (21).

## MATERIALS AND METHODS

The study was approved by the Committee on Ethics in Biomedical Research of the Heinrich Heine University, Düsseldorf, Germany. With the written informed consent of the parents, blood samples were taken from each 10 breast-fed and bottle-fed infants, respectively, at the age of six months of life (for further information about blood sampling, especially even at six weeks of age (21)). Although it was planned to collect blood samples from each 25 breast-fed and bottle-fed infants at four fixed times (6 weeks, 3 months, 6 months, and 12 months), the complete amount of blood samples were only available from each 10 breast-fed and bottle-fed infants at six weeks and six months of age, respectively, due to refusing blood sampling by many parents at 3 months and 6 months of age, whereas the collection of the blood samples of the 10 infants remaining in the study population at 12 months of age is nearly completed, and serum will be analyzed as soon as the collection has been finished. Three higher chlorinated PCB congeners (IUPAC nos. 138, 153, and 180), HCB, and DDE were determined with capillary gas chromatography, as described elsewhere (21). In addition, reliability was tested with gas chromatography-mass spectrometry. The laboratory successfully participated in nationwide interlaboratory quality assessments for OC determinations. Possible correlations of OC with personal data (table) were tested with a standard multivariate regression model. Differences between study groups were tested on mean differences with Wilcoxon´s test for independent samples.

## RESULTS

There were no differences between the study groups of breast-fed and bottle-fed infants with regard to their personal characteristics (Table 1). All infants were exclusively breast-fed from birth until the time of investigation (six months). They did not become any additional food, with exception of tea in some cases. The serum concentrations of the three PCB congeners 138, 153, and 180 as well as ΣPCB (sum of the three PCB congeners), HCB, and DDE were significantly (*p*<0.0001) higher in breast-fed than in bottle-fed infants at six weeks of age (table) as well as at six months of age (figure).

**Table 1:** Personal data and analytical results of breast- and bottle-fed infants in our study. Data in underlined italics indicate statistically significant differences both between breast- and bottle-fed infants and infants at six weeks and six months of age, respectively. 1not significant; 2 Environmental tobacco smoke; 3 Statistically significant (*p*<0.0001; Wilcoxons test for independent samples) differences between bottle- and breast-fed infants

Age at blood sampling	Breast-fed infants (n=10)				Bottle-fed infants (n=10)				
	6 weeks		6 months		6 weeks			6 months	

	**Personal data [Median (Range)]**								
Sex (n; female/male)	4/6			ns 1		5/5			
Gestational age (weeks)	39.0 (35-40)			ns		39.0 (37-40)			
Birth weight (grams)	3222.5 (2650-3920)			ns		3090.0 (2170-4165)			
Mother’s age (years)	28.0 (17-34)			ns		28.5 (18-36)			
% of smoking mothers	30%			ns		30%			
% of mothers exposed to ETS 2	40%			ns		30%			
Age at time of blood sampling (weeks)	5.6 (5.0-6.0)	26.3 (25.6-27.1)		ns		5.8 (5.2-5.8)			25.9 (25.2-26.8)
	**Analytic results [μg/L; Median (Range)]**								
PCB 138	0.40 (0.04-0.73)		0.72 (0.24-0.76)		0.093 (0.01-0.38)		0.073 (0.00-0.43)		
PCB 153	0.57 (0.05-0.98)		0.99 (0.18-1.40)		0.113 (0.01-0.50)		0.093 (0.01-0.50)		
PCB 180	0.33 (0.02-0.58)		0.58 (0.01-0.23)		0.043 (0.02-0.58)		0.023 (0.07-0.89)		
∑ PCB	1.19 (0.16-2.25)		2.28 (0.12-3.02)		0.293 (0.12-0.63)		0.183 (0.02-1.32)		
HCB	0.13 (0.04-0.52)		0.43 (0.16-0.74)		0.043 (0.02-0.21)		0.073 (0.01-0.12)		
DDE	1.05 (0.76-3.49)		1.90 (0.21-4.63)		0.183 (0.07-0.54)		0.193 (0.03-0.68)		

## DISCUSSIONS

**Figure 1:**
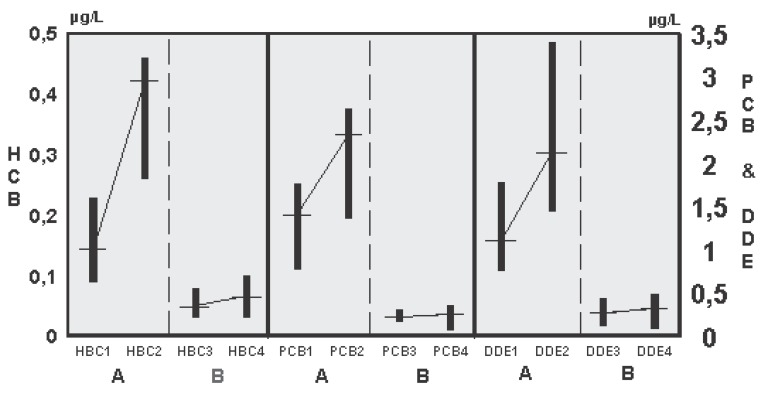
HCB, ∑PCB, and DDE concentrations (μg/L; median [crossbeam], 25th and 75th percentile by rank) of breast-fed (A) and bottle-fed (B) infants at the age of six weeks (1 and 3, respectively) and six months (2 and 4, respectively).

The results of the present study clearly demonstrate a significant increased body pollution of our infants with different harmful OC during lactational period. These results are remarkably as early as at six weeks of age with a further significant increase until the age of six months (Figure 1). Thereby, the OC concentrations of the bottle-fed infants correspond to the latest reference values of these substances in full-term neonates even at the age of six months (1), which may be attributed to the estimated daily OC intake of a bottle-fed infant of only 0.1 μg/kg body weight (2). On the other hand, the OC concentrations of the breast-fed infants are about 90% higher as early as at the age of six weeks when compared with their bottle-fed peers, and over and above nearly doubled further at the age of six months (table; figure). Any possible confounding factors, as gestational age, age of the mothers, and smoking behaviour of the parents could be excluded, since there were no differences between the study groups of breast- and bottle-fed infants with regard to these confounders (Table 1) (1, 21). Therefore, an infant, who is breast-fed for six months, today has a body pollution with OC that is as high as that measured in neonates born in Germany in the mid-1980s (1). Long-term breast-feeding leads to a dose- and time-dependent increase of PCBs, HCB, and DDE in children’s serum during the first 6 months of life. These disturbing findings make clear that the reduction of OC in our environment, e.g., a 70% to 90% decline in the prenatal uptake of these substances from the mid-1980s to the beginning of the 21st century (1), as a promising result of the prohibition and restriction of the use of OC in industrialised countries, will be obviously destroyed or even minimized by breast-feeding, in fact in a time-dependent manner. It, therefore, should be reconsidered again, if breast-feeding, despite all its advantages and all considerations, which were outlined in the Introduction of this paper, may possibly endure suffering any so far unknown health risks to our children. Although all National and International Committees engaged in the consideration of possible risks of breast-feeding due to OC have favoured breast-feeding for many years, future discussion about the recommendations of breast-feeding should incorporate the results of the present study to definitely answer the question, if the present recommendations for breast-feeding can still be maintained. To do this in a scientifically acceptable manner, further studies with larger study populations are urgently required to make sure, that breast-feeding can be unreservedly be recommended for all children in all countries of our world, i.e., for example, dependent on the availability of infant formulas in Third World countries.

This is of special importance, although - as outlined in the Introduction section - the mechanisms by which the pollution with different environmental pollutants prenatally or during infancy will produce harmful effects later in life, e.g., growth retardation, wheezing and asthma, and neurological impairment, are widely not understood today, because most studies, which were able to demonstrate harmful effects later in life, were performed in the 1960th to 1980th of the last century, the time span, when the body pollution was much higher as at the beginning of the 21st century, but corresponds to those OC-concentrations, which we were able to find in breast-fed infants at six weeks and six months of age in the present study today. Although it remains to be seen, whether the OC-concentrations found in breast-fed infants today will have any toxic effects to our children, the results are alarming, and further studies are urgently required to investigate the complex interaction of different environmental pollutants and the developing human organism (23).
